# Ameliorative Effects of a Combination of Baicalin, Jasminoidin and Cholic Acid on Ibotenic Acid-Induced Dementia Model in Rats

**DOI:** 10.1371/journal.pone.0056658

**Published:** 2013-02-20

**Authors:** Junying Zhang, Peng Li, Yanping Wang, Jianxun Liu, Zhanjun Zhang, Weidong Cheng, Yongyan Wang

**Affiliations:** 1 School of Basic Medical Sciences, Lanzhou University, Lanzhou, P. R. China; 2 State Key Laboratory of Cognitive Neuroscience and Learning, Beijing Normal University, Beijing, P. R. China; 3 The Laboratory Research Center of Xiyuan Hospital, China Academy of Chinese Medical Sciences, Beijing, P. R. China; 4 The Institute of Basic Clinical Medicine, China Academy of Chinese Medical Sciences, Beijing, P. R. China; “Mario Negri” Institute for Pharmacological Research, Italy

## Abstract

**Aims:**

To investigate the therapeutic effects and acting mechanism of a combination of Chinese herb active components, i.e., a combination of baicalin, jasminoidin and cholic acid (CBJC) on Alzheimer’s disease (AD).

**Methods:**

Male rats were intracerebroventricularly injected with ibotenic acid (IBO), and CBJC was orally administered. Therapeutic effect was evaluated with the Morris water maze test, FDG-PET examination, and histological examination, and the acting mechanism was studied with DNA microarrays and western blotting.

**Results:**

CBJC treatment significantly attenuated IBO-induced abnormalities in cognition, brain functional images, and brain histological morphology. Additionally, the expression levels of 19 genes in the forebrain were significantly influenced by CBJC; approximately 60% of these genes were related to neuroprotection and neurogenesis, whereas others were related to anti-oxidation, protein degradation, cholesterol metabolism, stress response, angiogenesis, and apoptosis. Expression of these genes was increased, except for the gene related to apoptosis. Changes in expression for 5 of these genes were confirmed by western blotting.

**Conclusion:**

CBJC can ameliorate the IBO-induced dementia in rats and may be significant in the treatment of AD. The therapeutic mechanism may be related to CBJC’s modulation of a number of processes, mainly through promotion of neuroprotection and neurogenesis, with additional promotion of anti-oxidation, protein degradation, etc.

## Introduction

Alzheimer’s disease (AD) has a very high morbidity in the senile population. It causes progressive impairment of cognitive performance, which develops into severe difficulty with household management and basic self-caring in the late stage [1]. At present, there is no satisfactory therapy for AD. Although several drugs have shown moderate amelioration of symptoms, none of them have sufficient potency to stop or reverse the pathological progression of AD [1].

**Figure 1:**
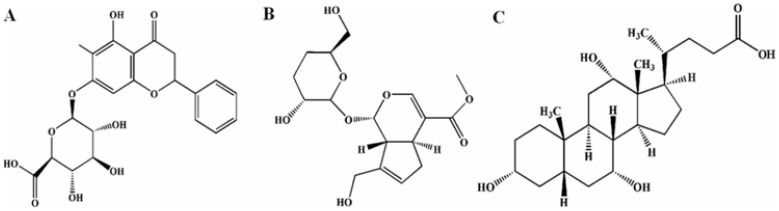
The structures of baicalin, jasminoidin and cholic acid. A. baicalin; B. jasminoidin; C. cholic acid.

Baicalin, jasminoidin, and cholic acid (figure 1) are the main active components of Qingkailing (QKL), one of the most well-known Chinese herb preparations. QKL is an aqueous preparation containing extracts of 7 herbs. It has shown an outstanding therapeutic effect on a broad spectrum of diseases, including high fever, coma, and acute inflammation, especially on stroke [2], [3]. However, as with other herbal preparations, QKL includes numerous unidentified compounds, which makes elucidating the therapeutic mechanism and controlling the preparation quality difficult. Additionally, these unidentified compounds can even cause adverse effects such as allergies and side-effects. Together, these inherent defects hinder the acceptance of herbal preparations including QKL by the mainstream medicine. In recent years, the concept of “active component combinations” has arisen in Chinese herbal therapeutics; this concept is proposed to identify the main active compounds in a formula and use them in combination instead of their parent herbs, thus keeping the advantages of the herbal combination, avoiding problems of uncontrolled composition, and making Chinese herb preparations qualified to meet the modern standards [4].

A series of pharmaceutical and pharmacodynamic studies have been conducted that identified more than 60 compounds in QKL and found 3 compounds, i.e. baicalin, jasminoidin, and cholic acid as the most active ones [4]. Baicalin, jasminoidin, and cholic acid are derived from 3 different herbs in QKL, Huangqin (the root of *Scutellaria baicalensis*), Zhizi (the fruit of *Gardenia jasminoides Ellis*), and Niuhuang (*Calculus bovis*), respectively; all of the 3 herbs have the function of “clearing heat and detoxifying”, according to Traditional Chinese Medicine theory. A combination of 3.75 mg baicalin, 18.75 mg jasminoidin and 5.25 mg cholic acid per kg of body weight was found to exert the greatest therapeutic effect in rat ischemic stroke models [5], [6].

Although this combination shows potential to replace QKL in stroke therapy, its effect on AD, another central nervous system disease with a number of similarities to stroke in its pathogenesis, has not yet been examined.

In the present study, the therapeutic potential and mechanism of action of this combination of baicalin, jasminoidin, and cholic acid (CBJC) on AD were evaluated in ibotenic acid (IBO)-injured rats. To our knowledge, this is the first report on the use of active component combinations of Chinese herbs in the treatment of AD, which may be significant in searching for effective drugs and improving Chinese herbal therapy for AD.

## Materials and Methods

This study was conducted in strict accordance with the recommendations in the Guide for the Care and Use of Laboratory Animals of the National Institutes of Health. The Institutional Review Board (IRB) of Beijing Normal University Animal Center approved the use of rats in this research project (January 1st, 2011–May 31st, 2011).

### Drugs and Reagents

Ibotenic acid (IBO) was purchased from Sigma (Atlanta, USA). Baicalin, jasminoidin and cholic acid were provided by Beijing University of Traditional Chinese Medicine (Beijing, China). 2-deoxy-2-(F-18)fluoro-D-glucose (FDG) was provided by the General Hospital of the People’s Liberation Army (Beijing, China). The rat cDNA microarray chip with 15,000 spots was purchased from Shanghai Biochip Inc. (Shanghai, China). Polyclonal anti-CRBP1 antibody was purchased from Pierce Thermo Fisher Scientific Inc. (Rockford, USA); polyclonal anti-EGF, anti-TRH, and anti-CBR1 antibodies were purchased from Abcam Inc. (Cambridge, UK); and polyclonal anti-ACE, monoclonal anti-β-actin, and horseradish peroxidase-conjugated anti-IgG antibodies were purchased from Santa Cruz Biotechnology Inc. (Santa Cruz, USA). Amersham ECL Plus western blotting detection reagents were purchased from GE Healthcare UK Ltd. (London, UK).

### Animal Surgery and Drug Administration

Male Sprague-Dawley rats weighing 250–300 g were purchased from the Experimental Animal Center of Beijing University. Animal treatment and care was conducted following NIH guidelines and was approved by the local animal care and use committee. The rats were randomly divided into 3 groups: control, IBO-model, and CBJC groups. Bilateral intracerebroventricular injection was performed under anesthesia induced by chloral hydrate (400 mg/kg, i.p.). The injection cannula was inserted into the lateral ventricle (3.0 mm posterior to the bregma, 2.0 mm lateral to the midline, and 2.8 mm below the dura), guided by a stereotaxic apparatus. A 1 *µ*L injection of IBO solution (10 g/L in sterile water, for the IBO-model and CBJC groups) or sterile water (for the control group) was performed slowly and evenly over 5 minutes using a Hamilton syringe and a microinjection pump. The solution of CBJC (1.25 mg/mL baicalin, 6.25 mg/mL jasminoidin, and 1.75 mg/mL cholic acid) was prepared in sterilized water and administered intragastrically at a dose of 3 mL/kg once a day from the third day after surgery until the rats were sacrificed. The rats in the control and IBO-model groups received water instead of CBJC.

### Morris Water Maze Test

The Morris water maze test began 1 month after the IBO injection. The water maze was a circular pool with a diameter of 120 cm and a height of 50 cm that was divided into four quadrants, filled with water and maintained at 24±1°C. Initially, a visible platform test was performed, which confirmed that there were no significant differences in sensory, motor or motivational activities between the groups. Then, hidden platform and reverse hidden platform tests were conducted in succession. For the hidden platform test, a round platform with a diameter of 9 cm was placed at the midpoint of the fourth quadrant, 2 cm below the water surface. A training trial was conducted once a day for 5 days. During each trial, the rats were placed in the water at a fixed position, opposite the platform and at the edge of the pool. The rats were allowed to swim freely until they escaped onto the platform. Swimming was recorded with a camera, and the escape latency time, swim distance, and swim speed were measured. The reverse hidden platform test was identical to the hidden platform test except the locations of the platform and the swim start were reversed; the reverse hidden platform trial lasted for 3 days.

### FDG Position-emission Tomography (FDG-PET) Examination

FDG-PET examination was performed on the day after the Morris water maze test finished. Rats were intravenously injected with FDG (diluted in saline, 3 mCi/kg). Forty minutes after injection, the rats were anesthetized by inhaling 5% isoflurane-95% O_2_ mixed gas, placed in a prone position and maintained under anesthesia with 1.5% isoflurane-98.5% O_2_ mixed gas. An Eplus166 micro PET scanner (manufactured by the Institute of High Energy Physics, Chinese Academy of Sciences, Beijing, China) was used to acquire the data. Images were reconstructed using the FORE plus OSEM method with a 128×128×63 matrix and saved in ANALYZE 7.5 format. Images were normalized, trimmed to remove data external to the brain, and smoothed with a 3.0×3.0×6.0 FWHM isotropic Gaussian kernel using a brain FDG-PET template created from the PET data of 24 normal rats and a statistical parametric mapping software (SPM2, Wellcome Department of Cognitive Neurology, London, UK). Finally, the images were subjected to voxel-based statistical analysis based on the general linear model.

### Histological Examination

After FDG-PET examination, 3 rats in each group were randomly selected for histological examination; the remaining animals were left for DNA microarray and western blotting analysis. The rats were anesthetized with chloral hydrate (400 mg/kg, i.p.) and their brains were perfused with saline followed by 4% formaldehyde via the ascending aorta. The perfused brains were then removed, post-fixed in 4% formaldehyde, and embedded in paraffin. The paraffin-embedded brains were cut into 5 µm coronal sections and subjected to hematoxylin and eosin (HE) staining. The histological morphology of the hippocampal CA1 region was observed under a light microscope.

### Tissue Preparation for DNA Microarray and Western Blotting

Rats were euthanized by decapitation, and their forebrains were rapidly removed, frozen in liquid nitrogen, and stored at −80°C until analysis. Each forebrain was divided into two parts by a vertical incision through the midline of the callosum; the left part was used for DNA microarray analysis, and the right part was used for western blotting.

### DNA Microarray

Tissues were homogenized in TRIzol, and the total RNA in each sample was extracted with chloroform and isopropyl alcohol. After purification with 75% alcohol, the RNA samples were qualified and quantified by agarose electrophoresis and UV spectrometry. RNA samples were reverse transcribed into cDNAs labeled with Cy3 (for samples in the control and CBJC groups) or Cy5 (for samples in the IBO-model group). The cDNA samples in the control and CBJC groups were randomly paired with samples from the IBO-model group. The paired samples were pooled and analyzed on the cDNA chip; the fluorescence ratio of Cy3 to Cy5 in each spot was measured according to the operator’s manual. A significant difference in Gene expression between groups was determined to exist when the average of the Cy3/Cy5 ratio was >1.6 or <0.6 and the *P* value for the comparison between the Cy3/Cy5 ratio and the Cy5/Cy5 ratio was <0.05.

### Western Blotting

The tissues were lysed in RIPA lysis buffer containing a cocktail of protease inhibitors (Roche Applied Science, Germany), and protein concentrations were determined using the BCA method. Aliquots containing 50 µg of protein in loading buffer (10% glycerol, 2% SDS, 60 mmol/L Tris-HCl, 0.01% bromophenol blue, and 100 mmol/L dithiothreitol, pH 6.8) were boiled for 5 minutes, subjected to SDS-PAGE and transferred to NC membranes. The levels of the proteins of interest and β-actin were detected using their corresponding primary antibodies and horseradish peroxidase-conjugated secondary antibodies at appropriate dilutions. Immunobands were lightened with Amersham ECL Plus western blotting detection reagents and imaged on X-ray film. The optical density (OD) of each protein band was quantified using the software Image J (NIH image, MD), and the OD value of each protein of interest was normalized to that of β-actin.

### Statistical Analysis

The time effect and group differences in the Morris water maze test were analyzed by one-way ANOVA with repeated measures followed by LSD post hoc test. Group differences in FDG-PET examination and DNA microarray were analyzed by two-tailed *t* tests for independent samples. Group differences in western blotting were analyzed by one-way ANOVA followed by LSD post hoc test. A *P* value <0.05 was considered statistically significant.

## Results

### Morris Water Maze Test

In the hidden platform test, the escape latency time was dependent on both the time effect (F_4,108_ = 18.178, *P<*0.001) and the group effect (F_2,27_ = 41.426, *P*<0.001); the control and CBJC groups escaped significantly faster than the IBO-model group (both *P*<0.001). A similar result was observed for the swim distance (F_4,108_ = 14.393 and *P<*0.001 for the time effect; F_2,27_ = 8.784 and *P*<0.001 for the group difference; and *P*<0.001 and *P*<0.05 for the comparisons of the control group and the CBJC group to the IBO-model group). Swim speed was not significantly different between the groups.

**Figure 2:**
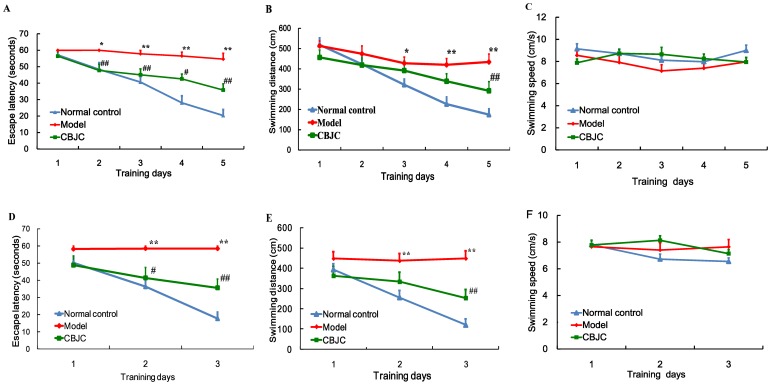
Effects of CBJC on cognition evaluated with the Morris water maze test. Escape latency time, swim distance and swim speed from the hidden platform test (A, B and C, respectively) and the reverse hidden platform test (D, E and F, respectively) are shown. The data are expressed as the means ± SEM (n = 10 in each group. Analysis was conducted by using one-way ANOVA with repeated measures followed by LSD post hoc test. * *P*<0.05, ** *P*<0.01, IBO-model group vs. control group; # *P*<0.05, ## *P*<0.01, CBJC group vs. IBO-model group).

In the reverse hidden platform test, the time effect and differences between the groups were both significant factors in the escape latency time (F_2,54_ = 12.607, *P<*0.001; F_2,27_ = 23.013, *P*<0.001); compared with the IBO-model group, the escape latency times in the control group and the CBJC group were significantly shorter (both *P*<0.001). A similar result was observed for the swim distance (F_2,54_ = 12.886 and *P<*0.001 for the time effect; F_2,27_ = 12.268 and *P*<0.001 for the group difference; and *P*<0.001 for the comparisons of the control group and the CBJC group to the IBO-model group). No significant differences in the swim speed were observed between groups (figure 2).

### FDG-PET Examination

**Figure 3:**
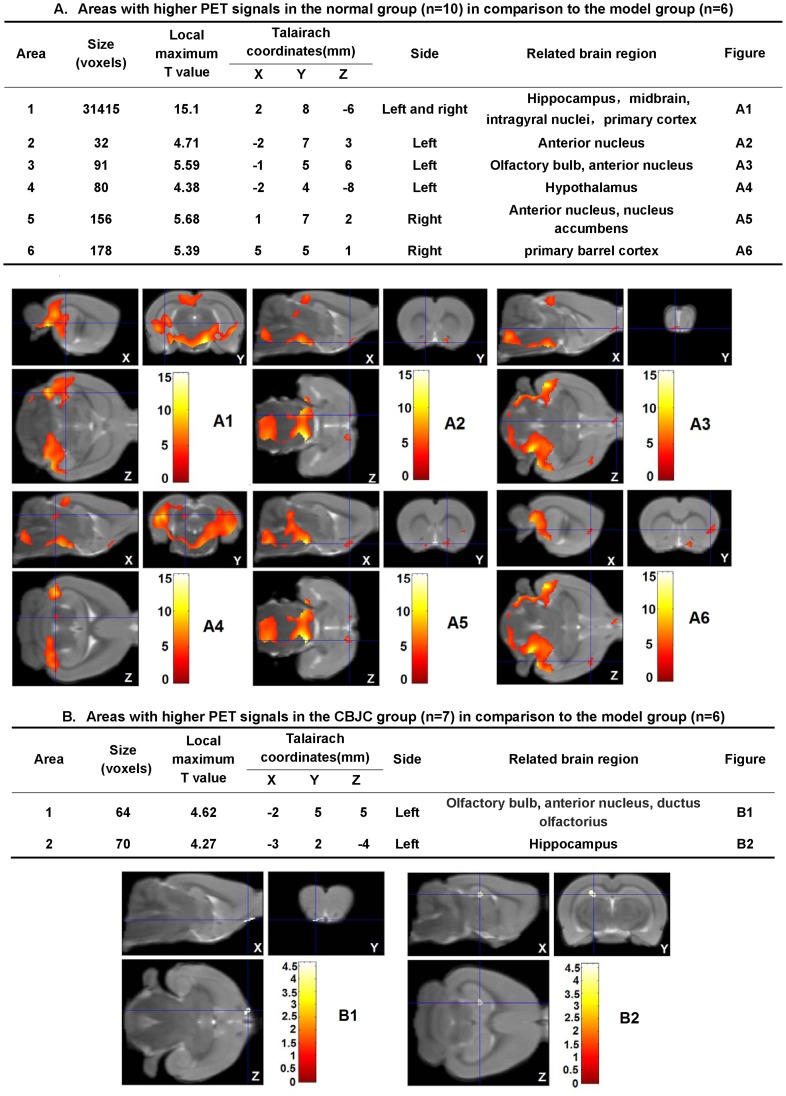
Effects of CBJC on the glucose-uptake in the brain. In the tables, the parameters of areas with significantly different **FDG-**PET signals between the normal group and the **IBO-**model group (A) **or** between the CBJC group and the **IBO-**model group (B) are shown (*P*<0.05). In the figures, the **FDG-**PET three-dimensional images of the areas listed in the tables are shown. In figure A3 and figure B1, the areas indicated by the intersection of blue lines are the common part of areas with decreased glucose-uptake in the **IBO-**model group in comparison to the normal group and that with increased glucose-uptake in the CBJC group in comparison to the **IBO-**model group. All the data were analyzed with 2-tailed *t* test for independent-samples.

Compared with the control group, significant decreases in FDG-PET signals were observed in the IBO-model group for numerous brain regions. The affected regions included the anterior nucleus, the hippocampus, the midbrain, the intragyral nuclei and the primary cortex on both sides, the olfactory bulb and the hypothalamus on the left side, and the nucleus accumbens and the primary barrel cortex on the right side (*P*<0.05). Compared with the IBO-model group, in the CBJC group, the decrease in the left olfactory bulb and the left anterior nucleus was significantly attenuated; additionally, a significant increase in the FDG-PET signal was observed in the left hippocampus (*P*<0.05, figure 3).

### Histological Examination of the Hippocampal CA1 Region

**Figure 4:**
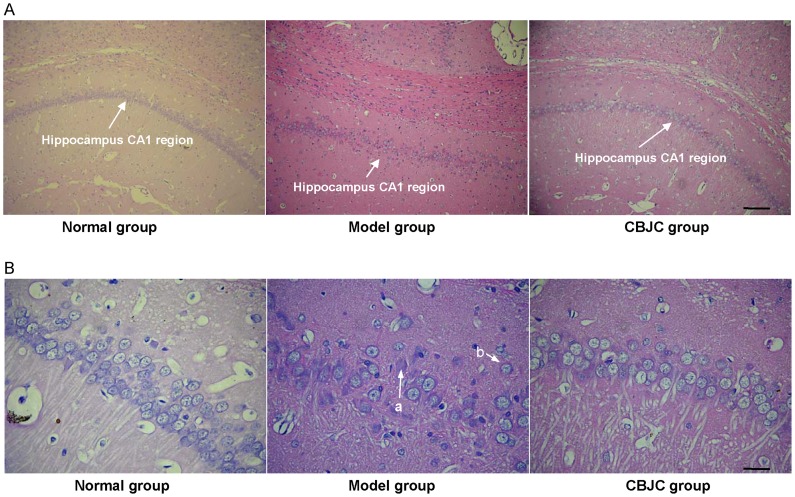
The histological morphology of the hippocampal CA1 region examined with HE staining. A: photomicrographs under **100×** magnification, scale bar = 40 µm; B: photomicrographs under **400×** magnification, scale bar = 10 µm. In the IBO-model group, neuron arrangement is disrupted, **severe lesions such as karyolysis (a) and eosinophilia (b) are observed in the nucleus and cytoplasm, and neuronal cell loss is noted.**

In the control group, neurons in the hippocampal CA1 region showed an orderly arrangement with no apparent abnormalities in cellular morphology. In the IBO-model group, neuron arrangement was disrupted with severe lesions in the nucleus and cytoplasm such as karyolysis and eosinophilia, and a neuronal cell loss was noted. In the CBJC group, these abnormalities were ameliorated (figure 4).

### Gene Expression in the Forebrain

Compared with the IBO-model group, in the CBJC group, the expression of 19 genes was significantly changed (*P*<0.05 or *P*<0.01). Of these 19 genes, 11 are related to neuroprotection and neurogenesis and the other 8 are related to anti-oxidation, protein degradation, cholesterol metabolism, angiogenesis, stress response, and apoptosis. Gene expression was increased in all cases except for the gene related to apoptosis (table 1).

**Table 1:** Gene expression changes induced by CBJC in the forebrain.

Function category	Gene	Ratio (M/C)	Ratio (CBJC/M)	Specific Function
Neuroprotection andneurogenesis	Cellular retinol binding protein 1 (CRBP1)	1.05±0.45	2.71±1.18^#^	See discussion.
	Epidermal growth factor (EGF)	1.16±0.08^*^	1.81±0.47^#^	See discussion.
	Fatty acid binding protein 7 (FABP7)	0.74±0.11^*^	1.84±0.21^##^	Promotes the up-take and storage of polyunsaturated fatty acids in neural cells. It is highly expressed in neuroepithelial cells and is essential for their maintenance during embryonic development. Up-regulated in proliferating neural progenitors after ischemia and in neural stem cells during their differentiation from embryonic stem cells [56]–[58].
	Insulin-like growth factor binding protein 5 (IGFBP5)	0.90±0.34	2.02±0.58^#^	Strongly enhances the activity of insulin-like growth factor which is a potent factor for neuron survival and genesis in neural tissue [59], [60].
	Major vault protein (MVP)	0.91±0.06^*^ (n = 3)	1.76±0.47^#^ (n = 3)	An intracellular transport protein, highly expressed in developing neurons and possibly having the function of transporting substances including mRNA from the neuron soma to the synapse [61].
	Midkine	0.91±0.23	2.17±0.90^#^	A heparin-binding and retinoic acid inducible growth factor, promoting the growth of neural precursor cells, protecting neurons from NMDA agonist-induced injury, and ameliorating brain ischemic injury by promoting neuronal regeneration [62]–[64].
	Potassium inwardly rectifying channel, subfamily J, member 13 (KCNJ13)	0.77±0.29	4.38±2.55^#^	Mediates an intracellular potassium current, decreasing the membrane potential and inhibiting depolarization [65].
	Retinoic acid induced 1 (RAI1)	1.09±0.03^**^	1.71±0.56^#^	A retinoic acid-inducible gene with an important role in the brain development, its loss results in defects in intelligence and locomotive activity, etc. [66].
	Thyrotropin releasing hormone (TRH)	0.79 (n = 1)	3.70±2.87^#^	See discussion.
	Transgelin	1.07±0.10	1.81±0.54^#^	An actin-binding protein, up-regulated in neuronal differentiation and regeneration [67], [68].
	Vasoactive intestinal peptide receptor 2 (VIPR2)	1.16±0.08^*^ (n = 3)	1.89±0.64^#^	Enhances the excitability of hippocampus CA1 neurons, protects neurons in excitotoxic injury, and promotes the proliferation of neural precursor cells [69]–[71].
Anti-oxidation	Carbonyl reductase 1 (CBR1)	1.14±0.33	1.91±0.52^#^	See discussion.
	Microsomal glutathione S-transferase 1 (MGST1)	0.64±0.19^*^	1.78±0.58^#^	Catalyzes the reduction of oxidants by glutathione and protects cells from multiple oxidant injuries [72].
Protein degradation	Angiotensin I converting enzyme (ACE)	0.66±0.30 (n = 2)	2.63±1.44^#^ (n = 3)	See discussion.
	RAD23 homolog B (S. cerevisiae) (RAD23B)	1.59±0.37^*^	2.69±0.64^#^	Transports proteins to the proteasome for degradation, possibly has a role in eliminating harmful proteins in neurodegenerative diseases [73].
Stress response	Cold inducible RNA binding protein (CIRBP)	0.91±0.34	1.91±0.88^#^	A stress-responsible gene, induced by hypothermia, hypoxia, DNA damage, etc. [74]. Stabilizes specific transcripts required for cell survival and has a protective effect under various stresses [75], [76].
Cholesterol metabolism	Lecithin cholesterol acyltransferase (LCAT)	0.95±0.21	1.80±0.66^#^	Esterifies cholesterol and facilitates the efflux of cholesterol from the brain [77].
Angiogenesis	Transmembrane 4 L six family member 5 (TM4SF5)	1.06±0.15	1.63±0.27^##^	Facilitates angiogenesis [78].
Apoptosis	Cyclin G1	1.72±0.71^*^ (n = 3)	0.57±0.18^##^	Facilitates neuronal apoptosis in AD [79].

Note: The table shows the expression data of the 19 genes that are significantly regulated by the CBJC treatment. The relative expression levels between the CBJC group and the IBO-model group, and between the IBO-model group and the control group of these 19 genes are shown (in the title line, M/C represents the ratio of the IBO-model group to the control group; CBJC/M represents the ratio of the CBJC group to the IBO-model group). The data are expressed as the means *±* SD, n* = *4 per group unless otherwise indicated. Analysis was conducted by using two-tailed *t* tests for independent-samples. *P<0.05, **P<0.01, IBO-model group vs. control group; ^#^ *P*<0.05, ^##^ *P*<0.01, CBJC group vs. IBO-model group.

### Verification for the Microarray Result by Western Blot

**Figure 5:**
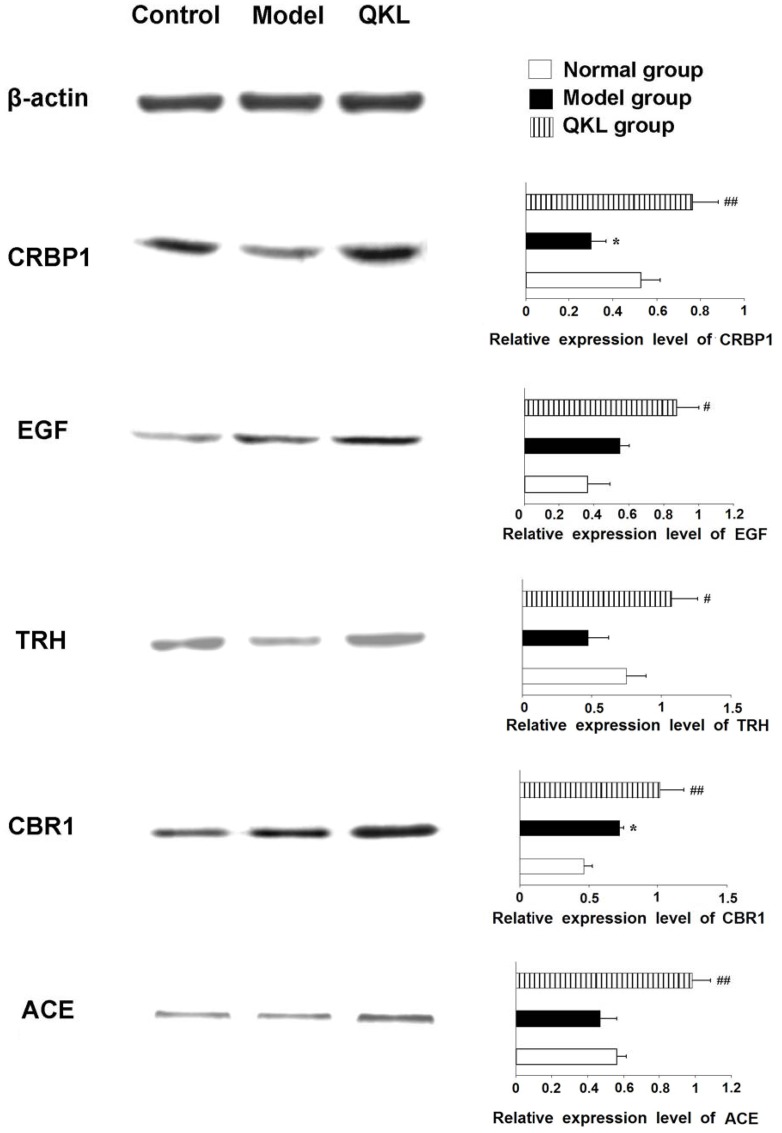
Protein expression of CRBP1, EGF, TRH, CBR1, and ACE in the forebrain. Representative western **blots** are shown **on** the left, the relative expression levels of the proteins of **interest** are shown **on** the right. **The** data are expressed **as the means** ± SD (n = 3 in each group. All the data were analyzed **by one-way** ANOVA followed by LSD post hoc test. *P<0.05, **IBO-**model group vs. control group; #*P*<0.05, ##*P*<0.01, CBJC group vs. model group).

TRH, EGF, CRBP1, CBR1, and ACE were selected for verification by western blot because previous studies have shown that they are more important in preventing AD in comparison to the others in the 19 genes significantly regulated by CBJC. The results showed that the protein expression levels of these 5 genes were significantly increased in the CBJC group compared with the IBO-model group (*P*<0.05 or *P*<0.01, figure 5), which was consistent with the results of the DNA microarray.

## Discussion

IBO is a potent NMDA receptor agonist that elicits severe injury and even death in neurons by inducing excessive calcium influx. Injecting IBO into the cerebroventricle leads to a direct exposure of the para-ventricular areas to this toxin; resulting in severe neuronal lesions in these regions, which may spread to the cerebral cortex. Intracerebral injection of IBO in animals such as rats and primates can elicit symptoms and pathological changes similar to those observed in human AD [7]–[9].

In the hidden platform and reverse platform tests, the escape latency time and the swim distance both significantly decreased as the training proceeded, indicating an ongoing spatial learning and memory in the rats; however, the extent of the decreases in the IBO-model group was significantly lower than that in the normal control and CBJC groups. As there was no significant difference in the swim speed among all the groups, this result indicates that the IBO injection elicited a significant impairment in spatial learning and memory and that this impairment was significantly ameliorated by the CBJC treatment. FDG-PET examination revealed a broad glucose-uptake decrease focused on the hippocampus, the basal nuclei, the cerebral cortex and the olfactory regions of the brain in the rats in the IBO-model group; this finding is indicative of hypofunction in the neurons in these regions, which is one of the most sensitive and characteristic features of AD [10]. CBJC significantly inhibited the decrease in the left olfactory brain and elicited a compensatory elevation in the left hippocampus. These two regions are both highly related to AD. The olfactory brain is the most sensitive region in AD, and it exhibits significant injuries even in very early stages of the disease [11]. The hippocampus is one of the pivotal regions of learning and memory, especially temporary memory, and its injury is recognized as a key procedure for AD [12]. Histological examination showed that the pathological changes induced by IBO in neurons in the hippocampus were inhibited by CBJC. These results indicate that CBJC is able to ameliorate the AD-like functional abnormalities and pathological changes in the IBO-injured rat model and therefore may be of use in retarding and even reversing the development of AD.

CRBP1 has a crucial role in maintaining retinoid contents; knockout of CRBP1 leads to an 80% decrease in retinoid contents in the body, while an up-regulation of CRBP1 results in a significant increase in body retinoid content [13], [14]. Interestingly, in the present study, besides CRBP1, the expressions of RAI1 and midkine were up-regulated by CBJC; they are both retinoid-inducible genes. These results strongly indicate that the content of retinoids in the forebrain might be significantly increased by the CBJC treatment, thus resulting in the activation of the retinoid pathway (the putative activation of CBJC on the retinoid pathway is shown in figure S1). The role of retinoids in AD is highlighted. Hypofunction of the retinoid pathway has been identified in the neocortex of AD patients and aged animals [15], [16]. The administration of retinoids shows a strong ameliorating effect on both transgenic and senile dementia mouse models [16], [17]. The anti-AD effect of retinoids is related to neuroprotection and neurogenesis. Retinol resists peroxidant injury in neurons and inhibits the formation and extension of Aβ in a cell-free system [18], [19]. More importantly, retinoids play a pivotal role in the modulation of neural precursor cells (NPCs). Retinoids are weak proliferating factors for NPCs; however, they are also strong neuronal differentiation-inducing factors [20], [21]. They elicit neuronal differentiation in a broad spectrum of naive cells including adult NPCs, neonatal neuroblasts, embryonic stem cells, and neural tumor cells [20]–[24]. In cultured NPCs, supplementation with retinoids can elevate the neuron differentiation ratio several times, even to nearly 100%, at the expense of glial differentiation [20]–[23].

EGF is a potent growth factor that is highly related to AD. The plasma content of EGF is significantly decreased in dementia patients, whereas supplementation with EGF strongly ameliorates cognitive defects in Aβ injured rats and senile rats [25]–[27]. One of the foundations of EGF’s anti-AD effect is its neuroprotective ability. EGF supports neuronal survival and outgrowth and protects neurons from multiple excitotoxic injuries [28]–[30]. Additionally, similarly to retinoids, EGF has an important role in the modulation of NPCs. The effects of EGF and retinoids are complementary. EGF is a potent proliferative factor for NPCs that strongly promote the mitogenesis of NPCs in vitro, in the normal adult brain, and in brains injured by Aβ, ischemia, and trauma [25], [31]–[35]; the differentiation-inducing effect of EGF is weak and exhibits a glial tendency [31], [32]. Interestingly, in the present study, the expression levels of EGF and CRBP1 were simultaneously increased by the CBJC treatment. This combined up-regulation might result in synergy in the re-genesis of neurons, with the up-regulation of EGF inducing more NPC proliferation and the up-regulation of CRBP1 increasing retinoid contents, which would lead to a higher neuronal differentiation ratio in the proliferated NPCs. The concurrent increase in the expression of EGF and CRBP1 might generate a greater rise in newborn neurons than could be achieved by any increase in the expression of either gene in isolation. The co-treatment of EGF and retinoids is a commonly used method for obtaining neurons from NPC [23], [33]; moreover, the combination of EGF and retinoids has been shown to have a synergic effect on neuron re-genesis in a neurodegenerative rat model [36].

TRH is a tripeptide that is distributed in neurons throughout the brain and acts as an endocrine hormone and a paracrine neuroregulator [37]. Administration of TRH to senescence-accelerated mice, fimbria-fornix lesioned rats, and AD patients was reported to ameliorate cognitive defects [38]–[40]. Moreover, the depletion of TRH in the culture medium of hippocampal neurons results in tau protein phosphorylation and neuron axonal retraction that is identical to the lesions observed in AD patients, indicating that the anti-AD effect of TRH is mainly related to its neuroregulatory role, which includes its ability to maintain neuron survival [41].

CBR1 strongly reduces the carbonyl in reactive lipid aldehydes, which are regarded as one of the most dangerous pathogenic factors in AD [42]. The overexpression of CBR1 prevents cells in culture from oxidant- and hypoxia-induced injuries [43], [44]; in *Drosophila*, overexpression of a human CBR1-resembled gene significantly attenuates neurodegeneration in the brain and the reduction in life span induced by oxidative stress, and prevents age-dependent defects in locomotor activity [45].

The effect of ACE on AD is to act as an Aβ-degrading protein, which is different from its traditional role in angiotensin metabolism. The genotype with a higher ACE expression was found at a significantly lower frequency in AD patients than in controls; in contrast, the genotype with a lower ACE expression was found at a significantly higher frequency [46]. Further studies found that Aβ deposition in the brain increased significantly when the activity of ACE was inhibited [47]. In vitro studies confirmed the ability of ACE to degrade Aβ and protect cells from Aβ-induced injury [48].

**Figure 6:**
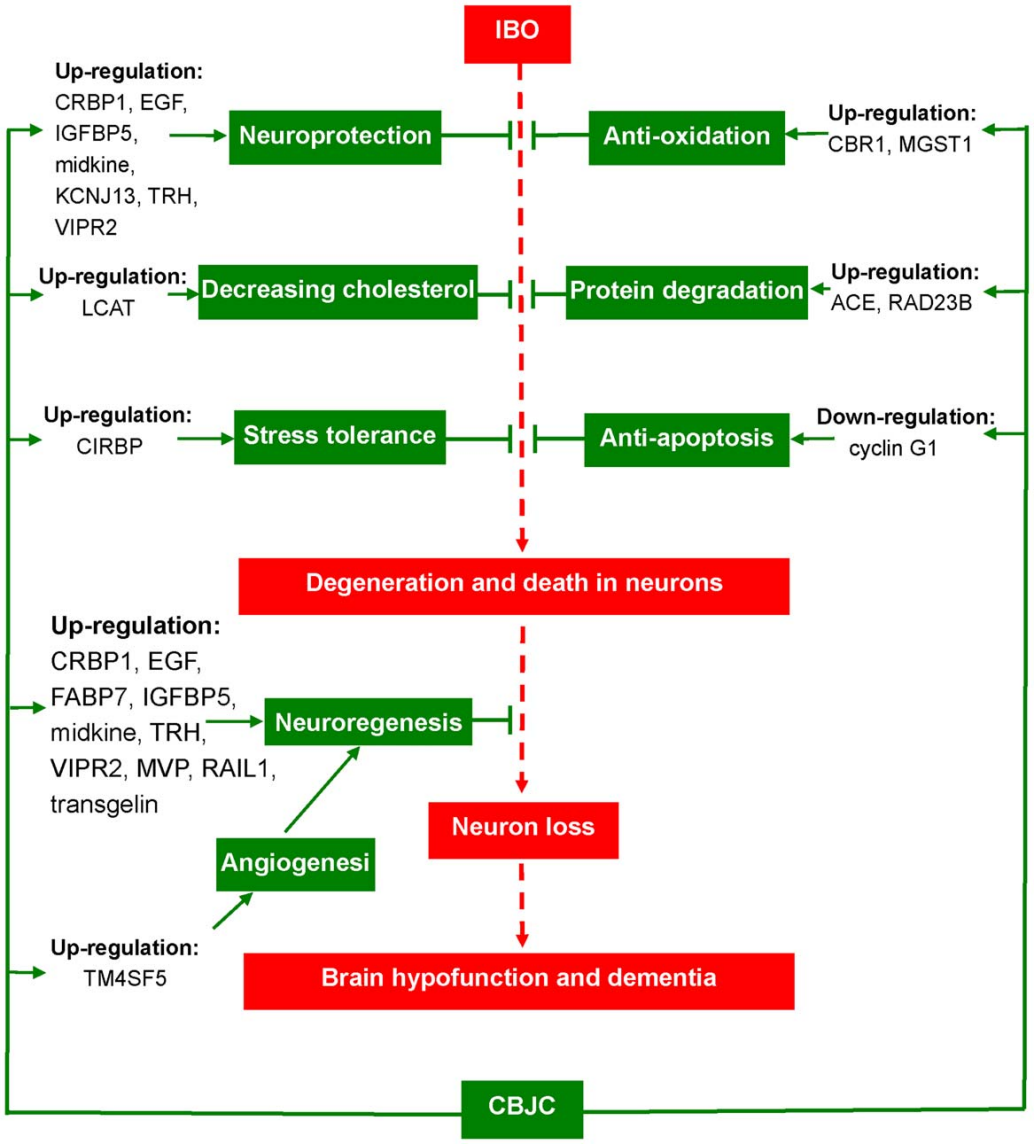
An ideograph of the putative therapeutic mechanism of CBJC on the AD model induced by IBO.

Generally, the gene expression profile showed that CBJC exerted its anti-AD effect by modulating a number of pathways in several AD-relevant areas, including neuroprotection and neurogenesis, oxidation, protein degradation, cholesterol metabolism, angiogenesis, stress, and apoptosis (the possible therapeutic mechanism of CBJC is summarized in figure 6). This multiple point-modulating capability is very suitable for treating complex diseases with multiple pathogenic factors, including AD. Pharmacodynamically, this capability is beneficial to achieve an amplified therapeutic effect through harmonious co-operation between the modulated points, such as the synergy of EGF and CRBP1 in the re-genesis of neurons.

Finally, the study raises the question of whether it is possible that CBJC can be further purified and replaced by one of its components. In our opinion, this outcome is unlikely as the 3 components of CBJC have distinct pharmacological characteristics. Baicalin has a strong anti-oxidation ability and a similar effect to retinoids in NPC modulation [49]–[51]; jasminoidin has a strong protective effect on neurons under a broad range of stresses [52]–[54]; and cholic acid strongly promotes the expression of growth factors in the brain [55]. Therefore, the combination could elicit all their effects together, acting like a poly-pill, and resulting in the multiple point-modulation that cannot be achieved by any single component treatment. CBJC has also shown a synergic effect on ischemic stroke [5].

Additionally, the result of DNA microarray offers a foundation and some clues for the future studies to further elucidate the mechanism of CBJC on AD. In these studies, the observations on the direct effects of CBJC on neurogenesis, neuronal oxidative stress, and Aβ degradation, etc. should be necessary.

In conclusion, the results of the present study suggest that CBJC is able to ameliorate the dementia induced by ibotenic acid in rats and may be of significant use in the treatment of AD. The therapeutic mechanism of CBJC may be related to its modulation of a number of pathways, principally involving the promotion of neuroprotection and neurogenesis, with additional involvement in anti-oxidation, protein degradation, etc.

## Supporting Information

Figure S1 **The activation of CBJC on the retinoid pathway inferred from the results of the present study.** CBJC treatment leads to an increase in the expression of CRBP1, which next promotes the intracellular uptake of retinol through binding with CRBP1. The intracellular retinol is then metabolized into retinal and retinoic acid, and these three are called “retinoids” collectively. Retinoids can exert direct neuroprotective effects, such as anti-oxidation and inhibiting the extension Aβ. Retinoids can also form a complex with RAR (retinoic acid receptors) and/or RXR (retinoid X receptors), inducing the expressions of genes with the abilities of neuroprotection and neurogenesis, such as RAI1 and midkine, through interacting with DNA.
(TIF)
