# Correction: Ameliorative Effects of a Combination of Baicalin, Jasminoidin and Cholic Acid on Ibotenic Acid-Induced Dementia Model in Rats

**DOI:** 10.1371/annotation/4588b718-f48e-4bf4-a387-e56f0a1be19e

**Published:** 2013-11-06

**Authors:** Junying Zhang, Peng Li, Yanping Wang, Jianxun Liu, Zhanjun Zhang, Weidong Cheng, Yongyan Wang

The first author, Junying Zhang, should be noted as contributing equally to this work along with the second and third authors, Peng Li and Yanping Wang. 

