# Supplementary figures and images for: Ameliorative Effects of a Combination of Baicalin, Jasminoidin and Cholic Acid on Ibotenic Acid-Induced Dementia Model in Rats
Source: PLoS One. 2013 Feb 20;8(2):e56658. doi: 10.1371/journal.pone.0056658 (PMC3577735; doi:10.1371/journal.pone.0056658)

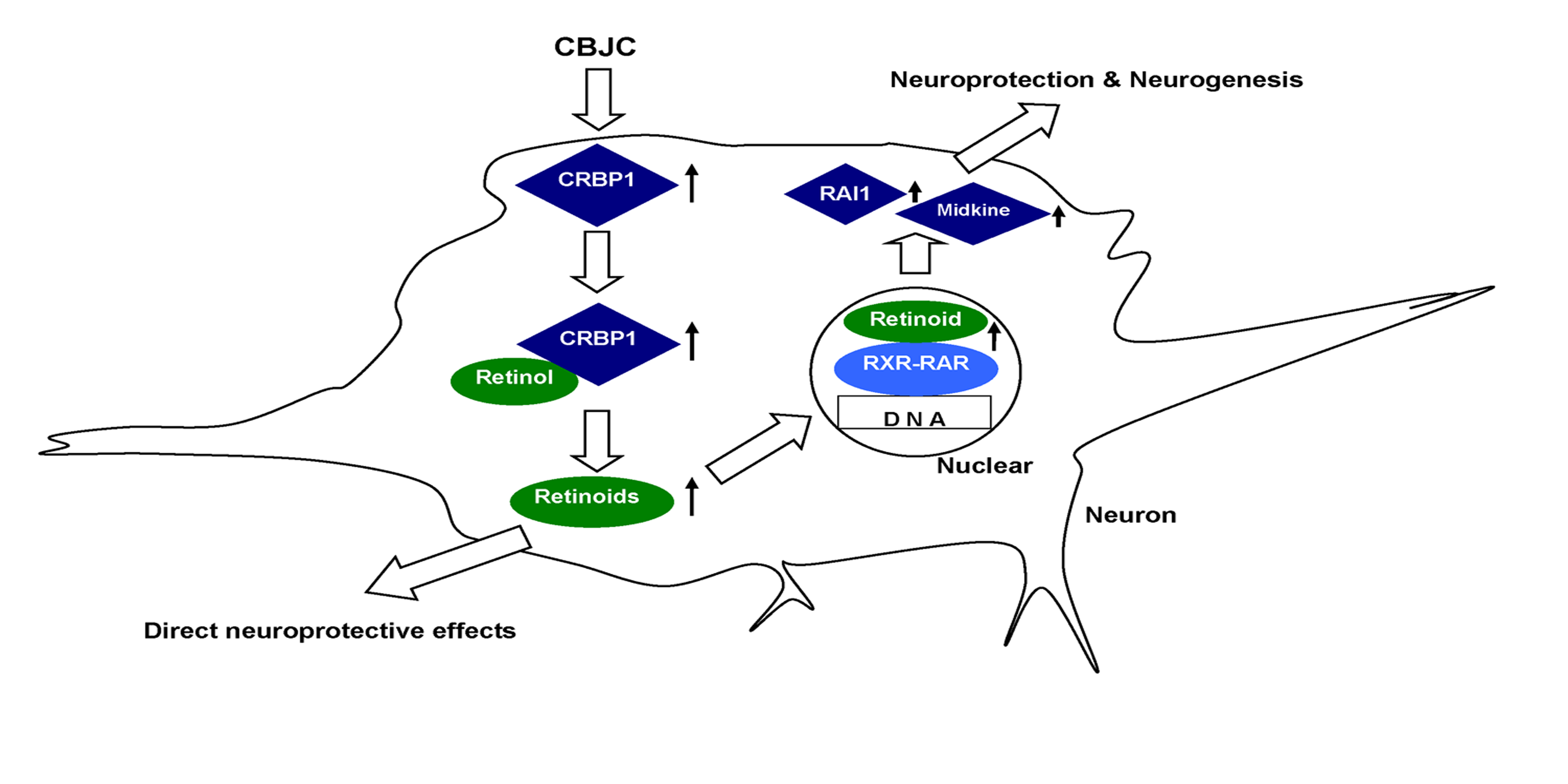

Supplement: Figure S1 — The activation of CBJC on the retinoid pathway inferred from the results of the present study. CBJC treatment leads to an increase in the expression of CRBP1, which next promotes the intracellular uptake of retinol through binding with CRBP1. The intracellular retinol is then metabolized into retinal and retinoic acid, and these three are called “retinoids” collectively. Retinoids can exert direct neuroprotective effects, such as anti-oxidation and inhibiting the extension Aβ. Retinoids can also form a complex with RAR (retinoic acid receptors) and/or RXR (retinoid X receptors), inducing the expressions of genes with the abilities of neuroprotection and neurogenesis, such as RAI1 and midkine, through interacting with DNA. (TIF) [file pone.0056658.s001.tif]
